# Wealth‐related inequalities in self‐reported health status in the United States and 14 high‐income countries

**DOI:** 10.1111/1475-6773.14366

**Published:** 2024-07-26

**Authors:** Ilias Kyriopoulos, Sara Machado, Irene Papanicolas

**Affiliations:** ^1^ Department of Health Policy London School of Economics and Political Science London UK; ^2^ LSE Health London School of Economics and Political Science London UK; ^3^ Department of Health Services, Policy and Practice Brown University School of Public Health Providence Rhode Island USA

**Keywords:** aging, Europe, health inequalities, United States, wealth

## Abstract

**Objective:**

To examine wealth‐related inequalities in self‐reported health status among older population in the United States and 14 European countries.

**Data Sources and Study Setting:**

We used secondary individual‐level data from Health and Retirement Survey (HRS) and the Survey of Health, Ageing, and Retirement in Europe (SHARE) in 2011 and 2019.

**Study Design:**

In this cross‐sectional study, we used two waves from HRS (wave 10 and 14) and SHARE (wave 4 and 8) to compare wealth‐related health inequality across countries, age groups, and birth cohorts. We estimated Wagstaff concentration indices to measure these inequalities across three age groups (50–59, 60–69, 70–79) and two birth cohorts (1942–1947, 1948–1953) in the US and 14 European countries.

**Data Collection/Extraction Methods:**

We performed secondary analysis of survey data.

**Principal Findings:**

Focusing on older population, we found evidence of wealth‐related inequalities in self‐reported health status across several high‐income countries, with the US demonstrating higher levels of inequality than its European counterparts. The magnitude of these inequalities with respect to wealth remained unchanged over the study period across all countries. Our findings also suggest that wealth‐related health inequalities differ at different stages of workforce engagement, especially in the United States. This could be explained either by potential redistributive effects of retirement or by uneven survivor effect, as less wealthy may drop out of the observations at a greater rate partly due to their poorer health.

**Conclusions:**

Wealth‐related inequalities in self‐reported health status are strong and persistent across countries. Our results suggest that there is meaningful variation across high‐income countries in health‐wealth dynamics that merits further investigation to better understand whether certain health or welfare systems are more equitable. They also highlight the need to consider social policy and wealth redistribution mechanisms as strategies for improving population health among the less wealthy, in the United States and elsewhere.

**What is known on this topic**

- There is high and rising wealth inequality in the United States.
- Social disadvantage and level of wealth are associated with health status.
- Wealth is an important factor to examine with regards to health outcomes, as it better reflects financial security and socioeconomic status compared with income, especially among older individuals.

**What this study adds**

- We measure wealth‐related health inequalities at different stages of workforce engagement and find a decline in the post‐retirement age in the US.
- Using established measures of socioeconomic inequality in health, this study shows that wealth‐related inequalities in self‐reported health status are higher in the United States than in European countries, across age groups and birth cohorts.
- Wealth‐related health inequalities remain unchanged over the study period across all countries.

## 1 INTRODUCTION

Income‐related health disparities are larger in the United States (US) than in most high‐income countries and among the largest in the world. While there is also high and rising wealth inequality in the US,^1^, ^2^ less is known about how the US compares to peer nations with regards to wealth‐related health inequalities. In the face of growing wealth inequalities, and wealth‐income ratios, the merits of a wealth tax are being debated by politicians, policy makers, and academics in the US and Europe.^3^, ^4^ As these debates ensue, it is important to understand the broader potential impacts of wealth inequalities, including how it may be related to high and growing health disparities across socioeconomic groups.^5^ Examining the level of wealth‐related health inequalities across countries may also provide insights to the extent nations differ in their ability to influence the link between health and wealth across countries.

For many reasons, wealth is an important factor to examine with regards to health outcomes, as it better reflects financial security and socioeconomic status compared with income, especially among older individuals.^6^, ^7^ First, while income represents the flow of money a person earns over a specified period, it is unclear how much of that income is truly available for spending on factors that may influence health, such as diet, housing, or care.^8^ Capturing the accumulated stock of assets and reserves, wealth is a more accurate representation of the resources an individual has at their disposal. It buffers the potential effect of temporary income loss, smooths consumption over time, and finances unpredictable household expenses.^9^ Furthermore, there are substantial differences in the amount of wealth, even among households who report similar income.

Second, most countries rely on income redistribution through taxation to tackle economic disparities, while wealth is taxed much less. Thus, the distribution of wealth is more chronically unequal. Wealth also appears to be a stronger predictor of health status than income across European countries and the US, contributing relatively more to health disparities.^10^, ^11^, ^12^ Furthermore, for those in later life, who are economically inactive and may have low or no income, wealth is an important source of purchasing power.^13^, ^14^ Thus, it may play a more important role in determining accessibility to health services, formal long‐term care, and even informal care, potentially acting as a self‐insurance mechanism and also mitigating the adverse stress‐related effects on health emerging from income insecurity.^15^

Last, wealth also better reflects economic opportunity, which is in turn an independent predictor of health.^16^ Although wealth has been increasingly acknowledged as a more suitable socioeconomic indicator to examine health inequities, especially for older ages,^9^, ^13^ little is known on how it contributes to health disparities across countries and over time. In addition, health inequalities with respect to wealth have not been examined from a comparative perspective in high‐income settings, as existing literature mainly focuses on analysis for single countries. More generally, there is little evidence on international comparisons of socioeconomic inequalities in health that also incorporate US.^17^ Building on previous work that examines the association between wealth and health across countries,^12^ we measure inequality in self‐reported health over the distribution of wealth, capturing the degree to which it differs across individuals ranked by wealth.^18^

In this study, we used harmonized survey data from the United States and 14 European countries to quantify wealth‐related health inequalities among older adults over time, using self‐reported health status as our main outcome. We set out to examine the following three questions: First, what is the magnitude of health inequalities with respect to wealth, among older adults in the US compared with their counterparts in other high‐income countries, in 2011 and 2019? Second, do wealth‐related health inequalities differ across countries for adults aged 50–59 (i.e., while working), 60–69 (i.e., during the later years of working life or first years of retirement), and 70–79 (i.e., where most adults have retired)? Third, do wealth‐related health inequalities among individuals from specific birth cohorts change as they age?

## 2 METHODS

### 2.1 Data

We used publicly available data from the RAND Health and Retirement Survey (HRS) and the Survey of Health, Ageing, and Retirement in Europe (SHARE). These surveys are conducted biennially, focus on individuals older than 50 years, and contain detailed information on several variables including health and wealth. To ensure comparability, we used the harmonized versions of the surveys, compiled by Gateway to Global Aging Data project of University of Southern California.^19^ We employed data from HRS Waves 10 and 14 and SHARE Waves 4 and 8, with most respondents interviewed in 2011 and 2019.

### 2.2 Age and cohort construction

We were interested in understanding how health‐wealth inequality varies across countries and time (time effect), and the degree of health‐wealth inequalities changes for people in different age groups, specifically pre‐ and post‐ retirement (age effect), or for different generations (cohort effect).

To explore the age effect, we first identified respondents in three specific age groups: 50–59, 60–69, and 70–79 years in 2011, and compared them with respondents that have the same age in 2019 (Table S1). We focused on these three age groups as the first consists of people who participate in the labor force, the second group is composed of older adults being in the later years of working life or going into retirement, and the latter will primary include retired individuals. Using these populations, we ensured that we compare the cross‐sections of individuals in the same age groups over time.

To explore the cohort effect, we concentrated on specific cohorts of individuals as they age. In doing so, we focused on participants born between 1942 and 1947 and compared the wealth‐related health inequality among them in 2011 and 2019, as they get older. We also considered a younger birth cohort, corresponding to those born from 1948 to 1953. More details on the definitions of the study population can be found in Table S1. The time effect was explored across both age groups and cohort groups by examining values in 2011 and 2019.

### 2.3 Key variables

Our determinant of interest was the net value of the total non‐housing wealth, due to its liquid nature as well as potential cohort‐specific differences in housing assets.^20^, ^21^ As a robustness check, we have also employed a measure of total wealth. All monetary values were adjusted to 2015 prices, using the Consumer Price Index (CPI). Our health outcome was based on individual's rating of their health status and was measured on a 5‐point scale (i.e., poor, fair, good, very good, excellent). Similar to previous studies, a binary variable was constructed taking the value of 1 for those having good, very good, or excellent health.^22^, ^23^, ^24^ Self‐reported health is a valid and reliable measure of health status,^25^ a strong predictor of mortality, and morbidity,^26^, ^27^ and has been widely employed in population health, social science, and clinical research^27^, ^28^ and the literature on health inequality measurement across countries.^25^, ^29^, ^30^

### 2.4 Measurement of health inequality and statistical analysis

Several approaches have been proposed for the measurement of socioeconomic inequalities in health.^14^, ^31^, ^32^ We quantify wealth‐related health inequalities using a concentration index (CI). In particular, we measure inequality in self‐reported health over the distribution of wealth, capturing the degree to which it differs across individuals ranked by wealth. CI is estimated with reference to the concentration curve, which illustrates the cumulative percentage of a health status variable in relation to the cumulative percentage of the population, with individuals ranked from the least wealthy to the wealthiest.^33^ In particular, CI is twice the area between the concentration curve and the 45‐degree line of equality, with zero values reflecting the absence of socioeconomic inequality in health. Contrary to the Gini index, CI quantifies inequality in one variable (i.e., health) based on its ranking relative to another (i.e., socioeconomic measure). A positive CI denotes that heath is more concentrated among wealthiest individuals, with higher index values reflecting greater levels of pro‐rich inequality. Given that there are several versions of the concentration index, our baseline analysis relies on Wagstaff (2005).^34^, ^35^

The analysis was performed in Stata 18, using the conindex command.^18^ Although we measured wealth‐related health inequalities for each country separately among those aged 60–69 and 70–79, we did not do so for the 50–59 age group due to reduced sample sizes in the last wave. We therefore reported concentration indices for the youngest age group for the US and Europe as a whole. When pooling the data from European countries, we have estimated the concentration index after adjusting for differences in purchasing power across countries. The relevant Stata package produces the results of *z*‐tests and *F*‐tests, assuming large sample and equal variances, respectively. We have tested the statistical significance of the differences in concentration indices using *z*‐tests and reported the resulting p‐values throughout the text.^18^ This study has received exemption by the Institutional Review Board of the Brown University School of Public Health.

## 3 RESULTS

### 3.1 Wealth and self‐reported health across age groups and birth cohorts

As shown in Table 1, our sample composed of 26,515 respondents aged 50–59, 35,619 respondents aged 60–69, and 30,852 respondents aged 70–79. In the younger age group, approximately 7 out of 10 respondents reported good, very good, or excellent health (“good health” throughout the text) in both United States and Europe. The share of individuals reporting good health decreased in the older age groups across all European countries, reaching 53% among those aged 70–79. However, this is not the case in the United States, where the share of respondents reporting good health was similar across all age groups. It is noteworthy that these percentages might be affected by a survival effect, in the sense that people with very poor health die at different rates across countries. From a comparative perspective, there was substantial variation in the share of people reporting good health across countries, with Eastern European countries consistently performing worse compared with the US and their Western European counterparts. For example, the proportion of respondents reporting good health in Estonia was 44%, 33%, and 19% among those aged 50–59, 60–69, and 70–79, respectively. On the contrary, more than 80% of respondents in Switzerland rated their health as good in all age groups examined. Similar findings were observed when focusing on respondents from different birth cohorts.

**TABLE 1:** Descriptive statistics of wealth and self‐reported health, by country and study population.

Self‐reported health	Sample: age group							Sample: birth cohort				
	50–59		60–69		70–79		Test *p*‐Value	1942–1947		1948–1953		Test *p*‐Value
	N	Good	N	Good	N	Good		N	Good	N	Good	
**Overall**	**26,515**	**69%**	**35,619**	**66%**	**30,852**	**57%**	**<0.001**	**19,267**	**62%**	**23,425**	**66%**	**<0.001**
**United States**	**8406**	**70%**	**7414**	**72%**	**8122**	**70%**	**0.016**	**3071**	**74%**	**4922**	**70%**	**<0.001**
**Europe**	**18,109**	**68%**	**28,205**	**65%**	**22,730**	**53%**	**<0.001**	**16,196**	**60%**	**18,503**	**65%**	**<0.001**
Austria	1552	76%	2243	74%	1830	63%	<0.001	1325	69%	1435	76%	<0.001
Germany	677	67%	1629	65%	1388	55%	<0.001	825	58%	1152	67%	<0.001
Sweden	323	76%	1483	77%	1578	70%	<0.001	1175	73%	981	77%	0.016
Spain	1044	71%	1676	60%	1678	48%	<0.001	1031	55%	1113	65%	<0.001
Italy	1073	76%	1912	65%	1697	46%	<0.001	1172	55%	1125	66%	<0.001
France	2010	72%	2648	70%	1952	56%	<0.001	1416	65%	1797	70%	<0.001
Denmark	1048	83%	1479	81%	1083	74%	<0.001	838	81%	943	81%	1
Switzerland	1307	86%	1940	85%	1458	80%	<0.001	1172	82%	1166	87%	0.003
Belgium	2082	75%	2316	73%	1608	65%	<0.001	1198	72%	1576	73%	0.55
Czech Republic	1685	66%	3077	66%	2310	56%	<0.001	2007	62%	1998	68%	<0.001
Poland	768	63%	1526	52%	944	33%	<0.001	661	39%	1024	53%	<0.001
Hungary	1065	48%	1436	48%	893	33%	<0.001	739	44%	1036	45%	0.77
Slovenia	1167	69%	1797	67%	1407	52%	<0.001	933	58%	1200	66%	<0.001
Estonia	2308	44%	3043	33%	2904	19%	<0.001	1704	29%	1957	34%	0.008

Wealth	Sample: age group							Sample: birth cohort				
	50–59		60–69		70–79		Test *p*‐value	1942–1947		1948–1953		Test *p*‐value
	Median	p25–p75	Median	p25–p75	Median	p25–p75		Median	p25–p75	Median	p25–p75	
**Overall**	**18.1**	**1.9–104.0**	**22.3**	**3.1–130.4**	**19.1**	**2.4–120.2**	**<0.001**	**21.9**	**3.49–132.8**	**21.5**	**2.8–118.8**	**0.12**
**United States**	**10.8**	**0.0–85.1**	**28.1**	**1.1–239.8**	**59.4**	**4.9–323.9**	**<0.001**	**71.3**	**6.6–388.8**	**18.9**	**0.1–172.9**	**<0.001**
**Europe**	**22.2**	**3.4–111.8**	**21.4**	**3.5–114.8**	**14.3**	**2.1–78.2**	**<0.001**	**18.8**	**3.2–104.4**	**22.1**	**3.43–111.6**	**0.042**
Austria	27.0	6.0–80.5	22.7	7.6–73.4	17.3	5.0–48.4	0.006	21.0	7.4–59.2	25.8	7.6–81.5	0.53
Germany	49.3	8.7–153.4	45.7	12.4–144.7	31.2	10.4–103.7	<0.001	34.1	10.9–106.2	44.7	12.4–136.3	0.05
Sweden	130.0	33.2–292.4	137.2	40.5–316.1	104.4	32.2–302.8	0.31	136.5	40.1–344.1	135.8	38.9–334.7	0.024
Spain	12.6	2.7–101.2	16.4	3.4–95.1	8.6	1.2–50.4	<0.001	11.2	2.5–73.3	17.2	3.4–95.1	0.49
Italy	15.3	4.2–62.5	13.7	4.0–57.2	11.7	3.4–48.6	<0.001	13.6	3.8–48.9	13.6	4.2–54.4	0.24
France	34.8	8.2–133.2	45.2	11.3–154.8	32.3	7.9–130.3	<0.001	45.2	11.3–168.3	42.7	11.3–153.4	0.06
Denmark	125.8	32.3–330.1	153.7	41.9–395.8	73.4	17.9–211.1	<0.001	97.9	27.2–265.5	147.5	44.5–395.8	<0.001
Switzerland	173.7	39.3–491.2	230.4	60.0–633.4	149.1	37.3–482.0	0.007	204.7	49.9–593.7	212.1	56.0–629.9	0.37
Belgium	71.7	18.5–213.2	85.8	19.7–266.4	46.5	12.5–217.9	0.002	63.8	14.0–247.8	84.8	24.0–265.1	0.06
Czech Republic	8.8	2.1–28.0	8.4	1.9–27.5	5.0	0.8–17.6	<0.001	6.6	1.6–21.8	9.6	2.3–30.5	<0.001
Poland	3.0	0.2–13.4	2.3	0.1–8.8	0.8	0.0–3.9	<0.001	1.1	0.0–4.7	2.3	0.1–7.3	0.026
Hungary	2.8	0.0–11.0	1.9	0.0–7.4	0.3	0.0–3.6	<0.001	1.5	0.0–6.6	1.8	0.0–8.8	0.11
Slovenia	10.3	2.7–38.2	8.3	1.8–31.6	6.0	1.0–23.7	<0.001	7.6	1.4–27.4	9.2	2.1–32.5	0.24
Estonia	6.3	0.5–55.9	4.8	0.6–37.5	2.5	0.4–15.5	<0.001	4.1	0.6–29.8	4.6	0.6–40.0	0.018

*Note*: Test *p*‐value refers to the *p*‐value of a Chi‐square test of equality of proportions (binary variables) or medians (continuous variables). The bold indicates aggregated values for the entire US and Europe as well as the overall sample.

Focusing on the sample characteristics for non‐housing wealth, some interesting patterns also emerged. First, older respondents in the US tended to accumulate more wealth compared with their younger counterparts. The median wealth among those aged 70–79 was more than twice the wealth of those aged 60–69 and more than five times higher than that of the youngest cohort. This was not the case for Europe, where wealth among respondents aged 70–79 was lower as compared to the younger age groups. Second, from a comparative perspective, wealth was lower in the United States than in most Western European countries among those aged 50–59 and 60–69, but this was not necessarily the case when focusing on respondents aged 70–79. Third, wealth accumulation was substantially lower among Eastern European countries and was decreasing with age.

### 3.2 Wealth‐related inequalities in self‐reported health, by age group

**FIGURE 1:**
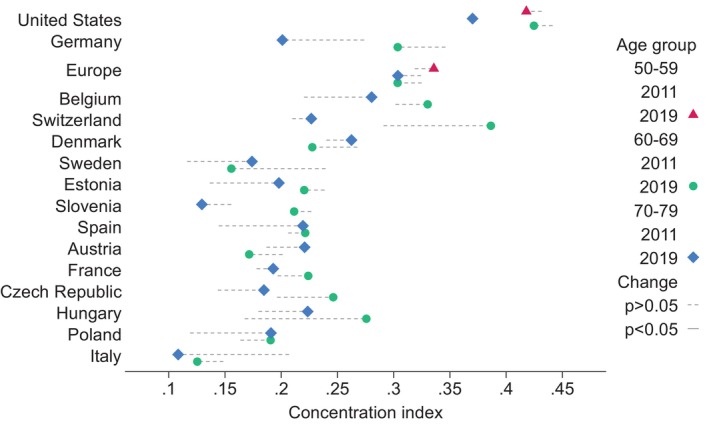
Estimates of the concentration index across age groups. Defined with reference to the concentration curve, the concentration index is a widely used measure to quantify socioeconomic inequality in health. In this case, a positive value of the concentration index indicates that the health variable is concentrated among better‐off individuals, thus implying disproportionate concentration of better health among the richest individuals. None of the changes in concentration index is statistically significant, as indicated by the dotted lines (*p*‐value >0.05).

Figure 1 presents the concentration indices by age group. Among individuals aged 60–69 and 70–79 concentration indices were statistically significant and positive for all countries examined, demonstrating pro‐rich health inequalities. We also measured wealth‐related inequalities in the 50–59 age group, and also reported significant wealth‐related health inequalities in Europe and the US. Wealth‐related health inequalities were consistently greater in the US than in the European countries for all years and age groups. Germany, Belgium, and Switzerland also had relatively higher concentration indices compared with the other study countries. There was almost no change for the estimated index for the same age groups in the US over time, particularly for the 70–79 age group. The magnitude of the concentration index for respondents aged 60–69 amounted to 0.441 (95% CI, 0.402–0.481) and 0.425 (95% CI, 0.386–0.463) in 2011 and 2019, respectively. Similarly, the concentration index in the 50–59 age group was 0.432 (95% CI, 0.399–0.464) in 2011 and 0.418 (95% CI, 0.378–0.458) in 2019. These differences in the magnitude of the concentration index over time were not statistically significant. While some concentration index estimates for the European countries changed over time, the differences were not statistically significant. The only exception was Italy among the older age group (70–79), for which wealth‐related inequalities in self‐reported health status decreased over the study period. The estimates of the concentration indices, along with their 95% confidence intervals, and the tests for differences between 2011 and 2019 for each country are presented in Tables S2–S4 for the 50–59, 60–69, and 70–79 age groups, respectively.

We also examined potential differences between the age groups for each country. We found that there is lower pro‐rich health inequality among those aged 70–79 as compared to those aged 60–69 in the US. In 2011, for example, the concentration index was 0.441 (95% CI, 0.402–0.481) among the 60–69 age group and 0.369 (95% CI, 0.337–0.402) in the older age group (70–79), with their difference between statistically significant (*p* < 0.01). We also reported statistically significant difference in the size of concentration index between the two age groups in 2019. As shown in Figure 1, concentration index is also lower among those aged 70–79 as compared to those aged 60–69 for most European countries. This difference was statistically significant for certain European countries, including Sweden, Belgium, and Estonia in 2011 and Germany and Switzerland in 2019. However, the differences between age groups in European countries should be interpreted with caution, as only the US is consistently an outlier in both 2011 and 2019 with the differences between the age groups being statistically significant. More details can be found in Tables S5 and S6.

### 3.3 Wealth‐related inequalities in self‐reported health, by birth cohort

**FIGURE 2:**
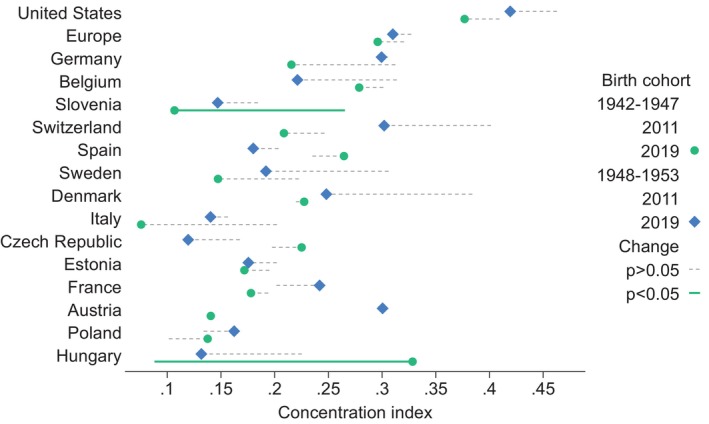
Estimates of the concentration index across birth cohorts. Defined with reference to the concentration curve, the concentration index is a widely used measure to quantify socioeconomic inequality in health. In this case, a positive value of the concentration index indicates that the health variable is concentrated among better‐off individuals, thus implying disproportionate concentration of better health among the richest individuals. The change in concentration index is significant (*p* < 0.05) in Hungary (increase) and Slovenia (decrease), as indicated by the green line. For the remaining countries, the changes in concentration index are not statistically significant, as indicated by the dotted lines (*p*‐value >0.05).

As pointed above, this analysis also examined whether wealth‐related health inequalities change over time for specific cohorts of individuals as they age. To do so, we focused on two birth cohorts: respondents born during 1942–1947 and 1948–1953. Similar to the findings for the age groups, we did not find evidence of changes in the size of wealth‐related inequalities in self‐reported health status over time in the US and most European countries (Figure 2). The only exceptions were observed among people born between 1942 and 1947 in Slovenia, Italy, and Hungary. In particular, it appears that there was a statistically significant reduction of the index in Slovenia and Italy, and an increase of wealth‐related inequalities in self‐reported health status in Hungary. Notably, the US lags behind its peers, with the concentration index being higher than that of its European counterparts for both cohorts. When we tested for differences across cohorts in 2011, we found that the cohort born later exhibits greater wealth‐related health inequality in Austria, Switzerland, and Hungary. In 2019, the cohort born later has lower wealth‐related health inequality in Hungary. For all other countries, there was not a significant difference. The detailed estimates for the concentration indices are presented in Tables S7–S9.

We have further compared wealth‐related health inequalities between the US and Europe, using two additional approaches. First, in addition to our baseline estimates, we have also estimated the concentration indices using a measure of total wealth instead of non‐housing wealth. As shown in Tables S10–S14, our results hold after changing the measure of wealth. Second, we repeated the estimates using a more objective variable capturing functional limitations associated with activities of daily living. These estimates, presented in Tables S15–S19, further confirm that wealth‐related health inequalities are greater in the US compared with Europe.

## 4 DISCUSSION

In this cross‐sectional analysis of survey data, we investigated how wealth‐related inequalities in self‐reported health among older adults compared in the US and other high‐income countries in 2011 and 2019. We found pro‐rich health inequality across all countries with the US exhibiting the greatest wealth‐related health inequalities of all study countries in both time periods and across all ages. Across all countries, the degree of wealth‐related health inequalities remained largely unchanged from 2011 to 2019. When exploring the wealth‐health gradient across different age groups pre‐ and post‐retirement, we found that several countries, and especially the US, exhibited lower degrees of inequality among the 70–79 age group. It is possible that the factors for this decrease differ across countries. We found little difference in wealth‐health gradient between two birth cohorts of baby‐boomers for most countries, although some evidence of increasing wealth‐related health‐disparity among the younger cohort in Austria, Switzerland, and Hungary.

Our findings have important implications for policy makers interested in understanding socioeconomic inequalities in health. First, we find that wealth‐related inequalities vary across countries with the US as an outlier in both time periods. This may reflect the higher degree of wealth inequity that exists in the United States coupled with well documented differences in coverage and cost‐related access barriers to healthcare.^36^ Healthcare coverage in many European countries is more comprehensive and includes lower amounts of out‐of‐pocket spending. This might result in improved access to care for the least wealthy both prior to Medicare eligibility, as well as after 65 where it has been documented that Americans incur higher out‐of‐pocket payments and are more likely to postpone or skip medical care due to financial barriers.^37^, ^38^ Second, most European countries have more egalitarian welfare states, which focus on social policies with health‐promoting spillovers, such as more generous unemployment and housing benefits. These policies may moderate the broader effects of social disadvantage on health and subsequently reduce wealth‐health inequalities.^39^ Third, higher wealth‐related health inequality could be attributed to accumulation of disadvantage throughout the life course among the poorer, due to the cumulative effects of long‐term exposure to stressors and risk factors. This explanations could relate to the role of stress, working environment, housing, or unhealthy behaviors over the life cycle, all of which are strong predictors of health.^40^, ^41^ This could serve as an additional explanation, particularly as many benefits in the United States over the life course are tied to employment. Another point relates to the role, design, and implementation of estate taxes, which might exhibit distinctive patterns in the way they influence long‐run wealth accumulation, distribution, and inequality across countries.^42^, ^43^, ^44^

We also find that there are differences in the magnitude of wealth‐related inequalities in self‐reported health status at different stages of workforce engagement, with a decline in inequalities in the US and several countries post‐retirement (70–79) as compared to the other groups. There are several potential factors that may contribute to this decline. One explanation may be that retirement has a redistributive effect, even for wealth. This could occur in countries where long‐term care or other social provisions are means tested, forcing wealthier individuals to tap into these resources while poorer households consume state provided services. Another explanation maybe that the disparity decreases because of an uneven survivor effect, where the least wealthy households die at younger ages at a greater rate due in part to their poorer health. The United States lags behind its peer countries in terms of life expectancy, which was 79 years in 2019 and about 5 years lower for the least educated,^45^ while there is also a widening gap between socioeconomic groups especially when compared to other high‐income countries.^46^, ^47^ Better understanding these factors can help policymakers to determine the extent to which policies may be able to influence the socioeconomic gradient earlier in the life course.

Our study contributes to a growing international literature examining social disparities in health across countries, using harmonized survey data from older adults.^48^, ^49^ While prior literature has documented that health of Americans is consistently worse than that of their European counterparts, with the differences being evident at all points of the SES distribution,^50^, ^51^ most studies explore health inequalities with regard to income^17^ and education.^52^ We contribute to this literature by exploring wealth as a measure of socioeconomic status. Using wealth as a proxy for living standards has several advantages compared with previously used socioeconomic measures, especially for older individuals.^6^, ^7^ Wealth can smooth the consumption patterns for and facilitates stable access to essential inputs for good health (e.g., medical care, education, housing, and food). Yet, only a few studies have explored health inequalities with respect to wealth, and even fewer have been comparative, despite differences in wealth inequalities across countries.^53^

Our findings are in line with studies showing that poorer Americans are in relatively worse position compared with their European counterparts.^41^ Another study revealed a qualitatively similar wealth‐health gradient in England and the US and highlighted the need for policies that concentrate on the broader social determinants of health.^13^ In this context, this study extended prior work by establishing the presence of strong and persistent wealth‐related health inequalities across countries, and by documenting differences across age groups and cohorts. In particular, we found that these patterns differ across countries with the US and some European countries seeing a reduction in wealth‐related inequality in self‐reported health status following retirement age. We also find that among a narrow set of younger cohorts, some countries see growing health‐related inequality while others see a decrease.

This study is not without limitations. First, we rely on a self‐reported measure self‐rather than objective health indicators. Although self‐reported health is considered as a valid and reliable measure of health that has been widely used in population health, social science, and clinical research, the results should be interpreted considering that this measure does not necessarily captures similar elements with other objective health indicators, such as biomarkers. In any case, we also estimated concentration indices using a more objective variable capturing functional limitations associated with activities of daily living. These estimates further confirm that wealth‐related health inequalities are greater in the US compared with Europe.

Second, we focus on specific population groups. As such, our results are not generalizable to other age groups, for which the level and trends of wealth‐related health inequalities might differ.

Third, although presenting results for each European country separately is interesting from a policy perspective, this might come at the expense of statistical power due to sample size issues for some countries. As such, some concentration index estimates might be surrounded by uncertainty. To address this potential issue, we have pooled European data and also arrived at a single estimate for Europe. These findings further confirm that wealth‐related health inequalities are consistently higher in the US.

Fourth, we did not provide country‐specific estimates of the concentration indices across European countries for the 50–59 age group, due to sample size limitations. We report relevant evidence for the US and Europe as a whole, but there might be significant differences across European countries.

Fifth, we are limited in the number and duration of birth cohorts we are able to observe and so can only draw limited findings about how wealth‐related health inequalities may be changing for individuals born later than 1953. It may be that we do not observe large differences between cohorts because we do not have enough data to observe cohorts that face more varied life circumstances.

Sixth, some interviews during the first and last waves took place in years earlier or later than 2011 and 2019, as HRS and SHARE waves take more than 1 year to be completed and are not conducted simultaneously.

Last, wealth at older ages is partly determined by health at younger ages. In particular, health influences productivity and labor market participation, thus affecting accumulated earnings over the lifetime. Poor health might also increase out‐of‐pocket payments for health, which are in turn wealth‐depleting. As such, health at younger age is correlated with wealth later in life, especially for chronic patients. This might lead to reverse causality when examining the potential impact of wealth and health. Nonetheless, this study does not examine the causal link between wealth and health, which is arguably confounded by many factors. Instead, using established health inequality measures, it aims to measure the degree of socioeconomic inequality in health after ranking individuals based on their wealth. As such, we abstain from drawing conclusions for the distinctive effect of wealth on health, as our study rather aims to measure the degree of health inequality between poor and wealthy.

## 5 CONCLUSIONS

Adopting a comparative perspective and focusing on older population, we found evidence of wealth‐related inequalities in self‐reported health across several high‐income countries, with the US demonstrating higher levels of inequality than its European counterparts. We found that several countries see a decrease in wealth‐related health inequality among cohorts that are post‐retirement age relative to working‐age individuals, although it is unclear if this is due to some redistributive effect that retirement has or survival bias. Finally, we see different patterns of the persistence of wealth‐related health inequalities across cohorts. Taken together, our results suggest there is meaningful variation across high‐income countries in health‐wealth dynamics that merits further investigation to better understand whether certain health or welfare systems are more equitable.

## FUNDING INFORMATION

The work in this article was not funded, although author IP was receiving funding from the Health Foundation during the time of the work.

## Supporting information


**Data S1.** Supporting information.
