# Supplementary material for: Wealth‐related inequalities in self‐reported health status in the United States and 14 high‐income countries
Source: Health Serv Res. 2024 Jul 26;59(6):e14366. doi: 10.1111/1475-6773.14366 (PMC11622277; doi:10.1111/1475-6773.14366)
Supplement: Supplementary file 1 — Data S1. Supporting information. [file HESR-59-0-s001.docx]

**Wealth-related inequalities in self-reported health status in the United States and 14 high-income countries**

Online Supplementary Material

**Table S1: Study population in 2011 and 2019**

| **Comparison** | **2011** | **2019** |
| --- | --- | --- |
| **Individuals aged** | 50-59 | 50-59 |
|  | 60-69 | 60-69 |
|  | 70-79 | 70-79 |
| **Individuals born between** | 1942-1947 | 1942-1947 |
|  | 1948-1953 | 1948-1953 |

**Table S2: Concentration index values using non-housing wealth, age group 50-59**

|  | CI (2011) | Lower bound (2011) | Upper bound (2011) | CI (2019) | Lower bound (2011) | Upper bound (2011) | P-value |
| --- | --- | --- | --- | --- | --- | --- | --- |
| United States | 0.432 | 0.399 | 0.464 | 0.418 | 0.378 | 0.458 | 0.604 |
| European countries | 0.302 | 0.284 | 0.321 | 0.337 | 0.290 | 0.385 | 0.181 |

**Note:** CI is the concentration index. Lower and upper bounds refer to 95% confidence interval. P-value is derived from statistical tests regarding the null hypothesis of equality of the index value between in 2011 and 2019. We did not provide country-specific estimates of the concentration indices across European countries for the 50-59 age group, due to sample size limitations.

**Table S3: Concentration index values using non-housing wealth, age group 60-69**

|  | CI (2011) | Lower bound (2011) | Upper bound (2011) | CI (2019) | Lower bound (2011) | Upper bound (2011) | P-value |
| --- | --- | --- | --- | --- | --- | --- | --- |
| United States | 0.441 | 0.402 | 0.481 | 0.425 | 0.386 | 0.463 | 0.555 |
| Austria | 0.201 | 0.141 | 0.262 | 0.172 | 0.055 | 0.288 | 0.656 |
| Germany | 0.346 | 0.255 | 0.437 | 0.304 | 0.231 | 0.376 | 0.474 |
| Sweden | 0.239 | 0.150 | 0.329 | 0.156 | 0.048 | 0.263 | 0.241 |
| Spain | 0.206 | 0.136 | 0.277 | 0.221 | 0.130 | 0.313 | 0.796 |
| Italy | 0.149 | 0.082 | 0.215 | 0.125 | 0.032 | 0.219 | 0.691 |
| France | 0.197 | 0.138 | 0.256 | 0.224 | 0.143 | 0.305 | 0.594 |
| Denmark | 0.268 | 0.159 | 0.376 | 0.228 | 0.125 | 0.330 | 0.600 |
| Switzerland | 0.291 | 0.201 | 0.381 | 0.386 | 0.272 | 0.501 | 0.200 |
| Belgium | 0.302 | 0.239 | 0.365 | 0.330 | 0.241 | 0.420 | 0.608 |
| Czech Republic | 0.196 | 0.147 | 0.246 | 0.246 | 0.159 | 0.334 | 0.330 |
| Poland | 0.164 | 0.079 | 0.249 | 0.191 | 0.112 | 0.269 | 0.648 |
| Hungary | 0.168 | 0.099 | 0.236 | 0.276 | 0.154 | 0.398 | 0.130 |
| Slovenia | 0.227 | 0.148 | 0.306 | 0.211 | 0.130 | 0.293 | 0.792 |
| Estonia | 0.238 | 0.186 | 0.291 | 0.220 | 0.147 | 0.294 | 0.697 |
| European countries | 0.308 | 0.291 | 0.325 | 0.300 | 0.277 | 0.323 | 0.591 |

**Note:** CI is the concentration index. Lower and upper bounds refer to 95% confidence interval. P-value is derived from statistical tests regarding the null hypothesis of equality of the index value between in 2011 and 2019.

**Table S4: Concentration index values using non-housing wealth, age group 70-79**

|  | CI (2011) | Lower bound (2011) | Upper bound (2011) | CI (2019) | Lower bound (2011) | Upper bound (2011) | P-value |
| --- | --- | --- | --- | --- | --- | --- | --- |
| United States | 0.369 | 0.337 | 0.402 | 0.370 | 0.325 | 0.415 | 0.976 |
| Austria | 0.187 | 0.120 | 0.253 | 0.221 | 0.126 | 0.315 | 0.564 |
| Germany | 0.274 | 0.174 | 0.374 | 0.201 | 0.126 | 0.276 | 0.254 |
| Sweden | 0.116 | 0.018 | 0.215 | 0.174 | 0.094 | 0.254 | 0.372 |
| Spain | 0.144 | 0.071 | 0.218 | 0.219 | 0.134 | 0.304 | 0.191 |
| Italy | 0.207 | 0.135 | 0.279 | 0.108 | 0.025 | 0.192 | 0.080 |
| France | 0.178 | 0.114 | 0.242 | 0.193 | 0.107 | 0.278 | 0.786 |
| Denmark | 0.240 | 0.116 | 0.364 | 0.262 | 0.163 | 0.362 | 0.783 |
| Switzerland | 0.210 | 0.110 | 0.309 | 0.227 | 0.118 | 0.335 | 0.821 |
| Belgium | 0.220 | 0.148 | 0.292 | 0.280 | 0.181 | 0.380 | 0.337 |
| Czech Republic | 0.144 | 0.077 | 0.210 | 0.185 | 0.112 | 0.257 | 0.414 |
| Poland | 0.119 | -0.005 | 0.243 | 0.191 | 0.093 | 0.288 | 0.373 |
| Hungary | 0.180 | 0.079 | 0.280 | 0.224 | 0.092 | 0.355 | 0.603 |
| Slovenia | 0.155 | 0.063 | 0.248 | 0.129 | 0.048 | 0.210 | 0.678 |
| Estonia | 0.136 | 0.067 | 0.206 | 0.198 | 0.114 | 0.282 | 0.267 |
| European countries | 0.304 | 0.284 | 0.323 | 0.297 | 0.275 | 0.319 | 0.663 |

**Note:** CI is the concentration index. Lower and upper bounds refer to 95% confidence interval. P-value is derived from statistical tests regarding the null hypothesis of equality of the index value between in 2011 and 2019.

**Table S5: Differences in concentration index using non-housing wealth between 70-79 and 60-69 age groups in 2011 and 2019**

|  | 2011 | p-value | 2019 | p-value |
| --- | --- | --- | --- | --- |
| United States | -0.072 | 0.006 | -0.055 | 0.070 |
| Austria | -0.014 | 0.752 | 0.049 | 0.519 |
| Germany | -0.072 | 0.295 | -0.103 | 0.053 |
| Sweden | -0.123 | 0.070 | 0.018 | 0.788 |
| Spain | -0.062 | 0.235 | -0.002 | 0.974 |
| Italy | 0.058 | 0.243 | -0.017 | 0.789 |
| France | -0.019 | 0.675 | -0.031 | 0.604 |
| Denmark | -0.027 | 0.745 | 0.035 | 0.632 |
| Switzerland | -0.081 | 0.235 | -0.160 | 0.048 |
| Belgium | -0.081 | 0.095 | -0.050 | 0.465 |
| Czech Republic | -0.053 | 0.212 | -0.062 | 0.288 |
| Poland | -0.045 | 0.561 | 0.000 | 0.996 |
| Hungary | 0.0122 | 0.844 | -0.052 | 0.569 |
| Slovenia | -0.071 | 0.251 | -0.082 | 0.161 |
| Estonia | -0.102 | 0.022 | -0.022 | 0.693 |
| European countries | -0.004 | 0.761 | -0.003 | 0.864 |

**Note:** P-value is derived from z-tests regarding the null hypothesis of equality of the index value between the group aged 70-79 and the group aged 60-69.

**Table S6: Differences in concentration index using non-housing wealth between 50-59 and other age groups in 2011 and 2019**

| Comparing 60-69 and 50-59 | 2011 | p-value | 2019 | p-value |
| --- | --- | --- | --- | --- |
| United States | 0.010 | 0.710 | 0.007 | 0.811 |
| European countries (PPP) | 0.005 | 0.685 | -0.037 | 0.164 |
| European countries | 0.006 | 0.638 | -0.032 | 0.231 |
|  |  |  |  |  |
| **Comparing 70-79 and 50-59** | **2011** | **p-value** | **2019** | **p-value** |
| United States | -0.062 | 0.008 | -0.048 | 0.119 |
| European countries | 0.001 | 0.931 | -0.040 | 0.132 |

**Note:** P-value is derived from z-tests regarding the null hypothesis of equality of the index value between the group aged 50-59 and the other age groups. We did not provide country-specific estimates of the concentration indices across European countries for the 50-59 age group, due to sample size limitations.

**Table S7: Concentration index values using non-housing wealth, birth cohort 1942-1947**

|  | CI (2011) | Lower bound (2011) | Upper bound (2011) | CI (2019) | Lower bound (2011) | Upper bound (2011) | P-value |
| --- | --- | --- | --- | --- | --- | --- | --- |
| United States | 0.409 | 0.350 | 0.468 | 0.377 | 0.308 | 0.446 | 0.483 |
| Austria | 0.141 | 0.061 | 0.221 | 0.141 | 0.017 | 0.264 | 0.995 |
| Germany | 0.312 | 0.188 | 0.437 | 0.216 | 0.115 | 0.316 | 0.237 |
| Sweden | 0.223 | 0.110 | 0.335 | 0.147 | 0.050 | 0.245 | 0.321 |
| Spain | 0.235 | 0.146 | 0.325 | 0.265 | 0.154 | 0.375 | 0.686 |
| Italy | 0.202 | 0.118 | 0.287 | 0.076 | -0.034 | 0.185 | 0.073 |
| France | 0.194 | 0.117 | 0.271 | 0.178 | 0.070 | 0.286 | 0.813 |
| Denmark | 0.220 | 0.068 | 0.371 | 0.227 | 0.097 | 0.358 | 0.939 |
| Switzerland | 0.246 | 0.133 | 0.360 | 0.209 | 0.076 | 0.341 | 0.671 |
| Belgium | 0.301 | 0.216 | 0.386 | 0.279 | 0.150 | 0.407 | 0.775 |
| Czech Republic | 0.198 | 0.134 | 0.261 | 0.225 | 0.136 | 0.314 | 0.622 |
| Poland | 0.101 | -0.019 | 0.222 | 0.138 | 0.001 | 0.274 | 0.697 |
| Hungary | 0.088 | -0.006 | 0.183 | 0.328 | 0.160 | 0.497 | 0.015 |
| Slovenia | 0.265 | 0.161 | 0.369 | 0.107 | 0.001 | 0.213 | 0.036 |
| Estonia | 0.195 | 0.124 | 0.267 | 0.172 | 0.063 | 0.281 | 0.726 |
| European countries | 0.303 | 0.280 | 0.325 | 0.289 | 0.261 | 0.318 | 0.474 |

**Note:** CI is the concentration index. Lower and upper bounds refer to 95% confidence interval. P-value is derived from statistical tests regarding the null hypothesis of equality of the index value between in 2011 and 2019.

**Table S8: Concentration index values using non-housing wealth, birth cohort 1948-1953**

|  | CI (2011) | Lower bound (2011) | Upper bound (2011) | CI (2019) | Lower bound (2011) | Upper bound (2011) | P-value |
| --- | --- | --- | --- | --- | --- | --- | --- |
| United States | 0.463 | 0.420 | 0.506 | 0.419 | 0.367 | 0.472 | 0.209 |
| Austria | 0.299 | 0.220 | 0.377 | 0.300 | 0.163 | 0.438 | 0.982 |
| Germany | 0.306 | 0.193 | 0.418 | 0.300 | 0.213 | 0.386 | 0.933 |
| Sweden | 0.306 | 0.182 | 0.430 | 0.192 | 0.075 | 0.309 | 0.190 |
| Spain | 0.204 | 0.112 | 0.295 | 0.180 | 0.070 | 0.290 | 0.747 |
| Italy | 0.156 | 0.064 | 0.249 | 0.140 | 0.029 | 0.252 | 0.827 |
| France | 0.202 | 0.130 | 0.274 | 0.242 | 0.144 | 0.339 | 0.515 |
| Denmark | 0.384 | 0.253 | 0.515 | 0.248 | 0.119 | 0.377 | 0.148 |
| Switzerland | 0.401 | 0.280 | 0.523 | 0.302 | 0.149 | 0.455 | 0.319 |
| Belgium | 0.314 | 0.239 | 0.388 | 0.221 | 0.106 | 0.336 | 0.186 |
| Czech Republic | 0.168 | 0.102 | 0.233 | 0.119 | 0.019 | 0.219 | 0.430 |
| Poland | 0.134 | 0.032 | 0.235 | 0.162 | 0.065 | 0.260 | 0.693 |
| Hungary | 0.225 | 0.145 | 0.305 | 0.132 | -0.012 | 0.276 | 0.265 |
| Slovenia | 0.184 | 0.087 | 0.281 | 0.147 | 0.049 | 0.245 | 0.594 |
| Estonia | 0.202 | 0.137 | 0.267 | 0.176 | 0.080 | 0.271 | 0.653 |
| European countries | 0.309 | 0.288 | 0.331 | 0.304 | 0.276 | 0.331 | 0.744 |

**Note:** CI is the concentration index. Lower and upper bounds refer to 95% confidence interval. P-value is derived from statistical tests regarding the null hypothesis of equality of the index value between in 2011 and 2019.

**Table S9: Differences in concentration index using non-housing wealth between 1948-1953 and 1942-1947 birth cohorts in 2011 and 2019**

|  | 2011 | p-value | 2019 | p-value |
| --- | --- | --- | --- | --- |
| United States | 0.054 | 0.150 | 0.043 | 0.335 |
| Austria | 0.158 | 0.006 | 0.160 | 0.090 |
| Germany | -0.007 | 0.937 | 0.084 | 0.216 |
| Sweden | 0.084 | 0.329 | 0.045 | 0.566 |
| Spain | -0.032 | 0.629 | -0.084 | 0.289 |
| Italy | -0.046 | 0.477 | 0.065 | 0.417 |
| France | 0.008 | 0.887 | 0.064 | 0.390 |
| Denmark | 0.164 | 0.108 | 0.021 | 0.826 |
| Switzerland | 0.155 | 0.068 | 0.093 | 0.365 |
| Belgium | 0.013 | 0.828 | -0.057 | 0.513 |
| Czech Republic | -0.030 | 0.519 | -0.106 | 0.122 |
| Poland | 0.032 | 0.687 | 0.025 | 0.774 |
| Hungary | 0.137 | 0.030 | -0.197 | 0.082 |
| Slovenia | -0.081 | 0.264 | 0.040 | 0.583 |
| Estonia | 0.007 | 0.889 | 0.004 | 0.960 |
| European countries | 0.007 | 0.671 | 0.014 | 0.480 |

**Note:** P-value is derived from z-tests regarding the null hypothesis of equality of the index value between the birth cohorts 1948-1953 and 1942-1947.

**Table S10: Concentration index values using total wealth, age group 50-59**

|  | CI (2011) | Lower bound (2011) | Upper bound (2011) | CI (2019) | Lower bound (2011) | Upper bound (2011) | P-value |
| --- | --- | --- | --- | --- | --- | --- | --- |
| United States | 0.402 | 0.369 | 0.435 | 0.426 | 0.386 | 0.466 | 0.367 |
| European countries | 0.303 | 0.284 | 0.322 | 0.336 | 0.289 | 0.384 | 0.209 |

**Note:** CI is the concentration index. Lower and upper bounds refer to 95% confidence interval. P-value is derived from statistical tests regarding the null hypothesis of equality of the index value between in 2011 and 2019. We did not provide country-specific estimates of the concentration indices across European countries for the 50-59 age group, due to sample size limitations.

**Table S11: Concentration index values using total wealth, age group 60-69**

|  | CI (2011) | Lower bound (2011) | Upper bound (2011) | CI (2019) | Lower bound (2011) | Upper bound (2011) | P-value |
| --- | --- | --- | --- | --- | --- | --- | --- |
| United States | 0.428 | 0.389 | 0.468 | 0.408 | 0.370 | 0.446 | 0.468 |
| Austria | 0.229 | 0.169 | 0.290 | 0.216 | 0.100 | 0.331 | 0.840 |
| Germany | 0.328 | 0.236 | 0.419 | 0.249 | 0.176 | 0.322 | 0.186 |
| Sweden | 0.243 | 0.154 | 0.333 | 0.212 | 0.105 | 0.319 | 0.662 |
| Spain | 0.194 | 0.123 | 0.264 | 0.204 | 0.112 | 0.295 | 0.863 |
| Italy | 0.147 | 0.080 | 0.213 | 0.179 | 0.087 | 0.272 | 0.573 |
| France | 0.184 | 0.125 | 0.243 | 0.197 | 0.115 | 0.278 | 0.805 |
| Denmark | 0.293 | 0.185 | 0.401 | 0.266 | 0.164 | 0.368 | 0.722 |
| Switzerland | 0.315 | 0.225 | 0.405 | 0.287 | 0.171 | 0.403 | 0.707 |
| Belgium | 0.322 | 0.260 | 0.385 | 0.388 | 0.299 | 0.476 | 0.237 |
| Czech Republic | 0.160 | 0.110 | 0.210 | 0.269 | 0.182 | 0.356 | 0.033 |
| Poland | 0.128 | 0.042 | 0.213 | 0.128 | 0.049 | 0.206 | 0.997 |
| Hungary | 0.230 | 0.162 | 0.298 | 0.288 | 0.166 | 0.410 | 0.417 |
| Slovenia | 0.240 | 0.161 | 0.319 | 0.159 | 0.078 | 0.241 | 0.166 |
| Estonia | 0.216 | 0.163 | 0.269 | 0.226 | 0.152 | 0.300 | 0.825 |
| European countries | 0.293 | 0.276 | 0.311 | 0.283 | 0.260 | 0.306 | 0.489 |

**Note:** CI is the concentration index. Lower and upper bounds refer to 95% confidence interval. P-value is derived from statistical tests regarding the null hypothesis of equality of the index value between in 2011 and 2019.

**Table S12: Concentration index values using total wealth, age group 70-79**

|  | CI (2011) | Lower bound (2011) | Upper bound (2011) | CI (2019) | Lower bound (2011) | Upper bound (2011) | P-value |
| --- | --- | --- | --- | --- | --- | --- | --- |
| United States | 0.351 | 0.318 | 0.383 | 0.354 | 0.309 | 0.399 | 0.913 |
| Austria | 0.184 | 0.117 | 0.250 | 0.206 | 0.111 | 0.300 | 0.707 |
| Germany | 0.313 | 0.213 | 0.412 | 0.190 | 0.115 | 0.265 | 0.053 |
| Sweden | 0.170 | 0.072 | 0.268 | 0.212 | 0.133 | 0.292 | 0.512 |
| Spain | 0.082 | 0.008 | 0.156 | 0.175 | 0.090 | 0.260 | 0.105 |
| Italy | 0.242 | 0.170 | 0.314 | 0.179 | 0.097 | 0.262 | 0.264 |
| France | 0.201 | 0.137 | 0.265 | 0.206 | 0.121 | 0.291 | 0.930 |
| Denmark | 0.277 | 0.153 | 0.400 | 0.293 | 0.194 | 0.392 | 0.841 |
| Switzerland | 0.238 | 0.139 | 0.338 | 0.219 | 0.110 | 0.328 | 0.797 |
| Belgium | 0.261 | 0.189 | 0.332 | 0.267 | 0.167 | 0.366 | 0.924 |
| Czech Republic | 0.064 | -0.003 | 0.131 | 0.190 | 0.117 | 0.263 | 0.013 |
| Poland | 0.054 | -0.076 | 0.184 | 0.163 | 0.064 | 0.261 | 0.191 |
| Hungary | 0.251 | 0.147 | 0.355 | 0.150 | 0.015 | 0.286 | 0.249 |
| Slovenia | 0.256 | 0.165 | 0.347 | 0.210 | 0.130 | 0.290 | 0.458 |
| Estonia | 0.176 | 0.107 | 0.245 | 0.195 | 0.111 | 0.279 | 0.732 |
| European countries | 0.269 | 0.249 | 0.289 | 0.274 | 0.252 | 0.296 | 0.734 |

**Note:** CI is the concentration index. Lower and upper bounds refer to 95% confidence interval. P-value is derived from statistical tests regarding the null hypothesis of equality of the index value between in 2011 and 2019.

**Table S13: Concentration index values using total wealth, birth cohort 1942-1947**

|  | CI (2011) | Lower bound (2011) | Upper bound (2011) | CI (2019) | Lower bound (2011) | Upper bound (2011) | P-value |
| --- | --- | --- | --- | --- | --- | --- | --- |
| United States | 0.409 | 0.350 | 0.468 | 0.355 | 0.286 | 0.424 | 0.244 |
| Austria | 0.200 | 0.121 | 0.280 | 0.186 | 0.063 | 0.308 | 0.845 |
| Germany | 0.295 | 0.170 | 0.420 | 0.192 | 0.091 | 0.293 | 0.208 |
| Sweden | 0.217 | 0.105 | 0.330 | 0.183 | 0.086 | 0.280 | 0.653 |
| Spain | 0.230 | 0.140 | 0.320 | 0.140 | 0.027 | 0.253 | 0.221 |
| Italy | 0.180 | 0.096 | 0.265 | 0.166 | 0.058 | 0.275 | 0.841 |
| France | 0.190 | 0.113 | 0.267 | 0.190 | 0.082 | 0.298 | 0.999 |
| Denmark | 0.205 | 0.053 | 0.356 | 0.326 | 0.197 | 0.454 | 0.234 |
| Switzerland | 0.272 | 0.159 | 0.384 | 0.201 | 0.068 | 0.333 | 0.425 |
| Belgium | 0.336 | 0.251 | 0.420 | 0.234 | 0.105 | 0.363 | 0.197 |
| Czech Republic | 0.181 | 0.118 | 0.245 | 0.189 | 0.100 | 0.279 | 0.890 |
| Poland | 0.082 | -0.039 | 0.203 | 0.185 | 0.048 | 0.322 | 0.270 |
| Hungary | 0.194 | 0.099 | 0.288 | 0.231 | 0.054 | 0.407 | 0.716 |
| Slovenia | 0.290 | 0.187 | 0.394 | 0.235 | 0.131 | 0.339 | 0.459 |
| Estonia | 0.175 | 0.103 | 0.246 | 0.185 | 0.076 | 0.294 | 0.876 |
| European countries | 0.287 | 0.265 | 0.310 | 0.265 | 0.237 | 0.294 | 0.236 |

**Note:** CI is the concentration index. Lower and upper bounds refer to 95% confidence interval. P-value is derived from statistical tests regarding the null hypothesis of equality of the index value between in 2011 and 2019.

**Table S14: Concentration index values using total wealth, birth cohort 1948-1953**

|  | CI (2011) | Lower bound (2011) | Upper bound (2011) | CI (2019) | Lower bound (2011) | Upper bound (2011) | P-value |
| --- | --- | --- | --- | --- | --- | --- | --- |
| United States | 0.436 | 0.393 | 0.480 | 0.400 | 0.348 | 0.452 | 0.295 |
| Austria | 0.246 | 0.168 | 0.325 | 0.271 | 0.133 | 0.410 | 0.760 |
| Germany | 0.343 | 0.232 | 0.455 | 0.253 | 0.166 | 0.341 | 0.213 |
| Sweden | 0.277 | 0.152 | 0.402 | 0.266 | 0.150 | 0.382 | 0.899 |
| Spain | 0.207 | 0.116 | 0.298 | 0.206 | 0.097 | 0.315 | 0.993 |
| Italy | 0.148 | 0.055 | 0.241 | 0.230 | 0.120 | 0.340 | 0.266 |
| France | 0.202 | 0.130 | 0.274 | 0.226 | 0.128 | 0.323 | 0.700 |
| Denmark | 0.359 | 0.228 | 0.491 | 0.279 | 0.151 | 0.408 | 0.393 |
| Switzerland | 0.441 | 0.319 | 0.562 | 0.280 | 0.126 | 0.433 | 0.106 |
| Belgium | 0.321 | 0.246 | 0.395 | 0.277 | 0.163 | 0.391 | 0.530 |
| Czech Republic | 0.137 | 0.072 | 0.203 | 0.183 | 0.083 | 0.282 | 0.456 |
| Poland | 0.117 | 0.015 | 0.219 | 0.091 | -0.007 | 0.190 | 0.720 |
| Hungary | 0.288 | 0.209 | 0.367 | 0.191 | 0.047 | 0.335 | 0.246 |
| Slovenia | 0.173 | 0.076 | 0.271 | 0.135 | 0.038 | 0.233 | 0.588 |
| Estonia | 0.224 | 0.159 | 0.288 | 0.208 | 0.113 | 0.303 | 0.787 |
| European countries | 0.301 | 0.280 | 0.323 | 0.285 | 0.258 | 0.313 | 0.365 |

**Note:** CI is the concentration index. Lower and upper bounds refer to 95% confidence interval. P-value is derived from statistical tests regarding the null hypothesis of equality of the index value between in 2011 and 2019.

**Table S15: Concentration index values using non-housing wealth, age group 50-59 (for limitations in ADLs)**

|  | CI (2011) | Lower bound (2011) | Upper bound (2011) | CI (2019) | Lower bound (2011) | Upper bound (2011) | P-value |
| --- | --- | --- | --- | --- | --- | --- | --- |
| United States | -0.399 | -0.443 | -0.355 | -0.394 | -0.447 | -0.342 | 0.900 |
| European countries | -0.228 | -0.264 | -0.191 | -0.253 | -0.336 | -0.169 | 0.596 |

**Note:** CI is the concentration index. Lower and upper bounds refer to 95% confidence interval. P-value is derived from statistical tests regarding the null hypothesis of equality of the index value between in 2011 and 2019. We did not provide country-specific estimates of the concentration indices across European countries for the 50-59 age group, due to sample size limitations.

**Table S16: Concentration index values using non-housing wealth, age group 60-69 (for limitations in ADLs)**

|  | CI (2011) | Lower bound (2011) | Upper bound (2011) | CI (2019) | Lower bound (2011) | Upper bound (2011) | P-value |
| --- | --- | --- | --- | --- | --- | --- | --- |
| United States | -0.368 | -0.419 | -0.317 | -0.358 | -0.407 | -0.309 | 0.786 |
| Austria | -0.301 | -0.406 | -0.195 | -0.280 | -0.477 | -0.083 | 0.855 |
| Germany | -0.400 | -0.563 | -0.238 | -0.266 | -0.397 | -0.135 | 0.206 |
| Sweden | -0.198 | -0.336 | -0.059 | -0.016 | -0.205 | 0.173 | 0.129 |
| Spain | -0.211 | -0.342 | -0.079 | -0.090 | -0.267 | 0.087 | 0.284 |
| Italy | -0.386 | -0.530 | -0.242 | -0.084 | -0.311 | 0.142 | 0.028 |
| France | -0.301 | -0.410 | -0.191 | -0.148 | -0.283 | -0.014 | 0.085 |
| Denmark | -0.352 | -0.555 | -0.148 | -0.213 | -0.387 | -0.040 | 0.309 |
| Switzerland | -0.276 | -0.444 | -0.108 | -0.419 | -0.616 | -0.222 | 0.277 |
| Belgium | -0.247 | -0.335 | -0.159 | -0.378 | -0.505 | -0.251 | 0.096 |
| Czech Republic | -0.120 | -0.213 | -0.026 | -0.267 | -0.389 | -0.146 | 0.059 |
| Poland | -0.039 | -0.165 | 0.087 | -0.148 | -0.270 | -0.026 | 0.224 |
| Hungary | -0.215 | -0.329 | -0.100 | -0.245 | -0.498 | 0.008 | 0.831 |
| Slovenia | -0.132 | -0.284 | 0.020 | -0.112 | -0.270 | 0.047 | 0.857 |
| Estonia | -0.160 | -0.232 | -0.087 | -0.247 | -0.371 | -0.123 | 0.232 |
| European countries | -0.241 | -0.272 | -0.210 | -0.213 | -0.253 | -0.172 | 0.276 |

**Note:** CI is the concentration index. Lower and upper bounds refer to 95% confidence interval. P-value is derived from statistical tests regarding the null hypothesis of equality of the index value between in 2011 and 2019.

**Table S17: Concentration index values using non-housing wealth, age group 70-79 (for limitations in ADLs)**

|  | CI (2011) | Lower bound (2011) | Upper bound (2011) | CI (2019) | Lower bound (2011) | Upper bound (2011) | P-value |
| --- | --- | --- | --- | --- | --- | --- | --- |
| United States | -0.334 | -0.373 | -0.295 | -0.302 | -0.356 | -0.247 | 0.341 |
| Austria | -0.209 | -0.306 | -0.112 | -0.179 | -0.325 | -0.033 | 0.737 |
| Germany | -0.367 | -0.512 | -0.221 | -0.331 | -0.436 | -0.227 | 0.700 |
| Sweden | -0.168 | -0.322 | -0.014 | -0.139 | -0.261 | -0.017 | 0.774 |
| Spain | -0.203 | -0.301 | -0.105 | -0.131 | -0.272 | 0.011 | 0.412 |
| Italy | -0.317 | -0.419 | -0.214 | -0.241 | -0.378 | -0.104 | 0.388 |
| France | -0.201 | -0.296 | -0.107 | -0.070 | -0.195 | 0.055 | 0.099 |
| Denmark | -0.172 | -0.352 | 0.009 | -0.136 | -0.282 | 0.011 | 0.763 |
| Switzerland | -0.053 | -0.210 | 0.104 | -0.179 | -0.364 | 0.005 | 0.305 |
| Belgium | -0.115 | -0.204 | -0.026 | -0.202 | -0.325 | -0.078 | 0.265 |
| Czech Republic | -0.135 | -0.233 | -0.036 | -0.106 | -0.201 | -0.010 | 0.679 |
| Poland | -0.059 | -0.193 | 0.074 | -0.204 | -0.335 | -0.073 | 0.129 |
| Hungary | -0.125 | -0.241 | -0.008 | -0.274 | -0.455 | -0.092 | 0.176 |
| Slovenia | 0.008 | -0.125 | 0.142 | -0.077 | -0.198 | 0.044 | 0.353 |
| Estonia | -0.148 | -0.211 | -0.086 | -0.152 | -0.250 | -0.054 | 0.951 |
| European countries | -0.195 | -0.224 | -0.167 | -0.185 | -0.219 | -0.152 | 0.650 |

**Note:** CI is the concentration index. Lower and upper bounds refer to 95% confidence interval. P-value is derived from statistical tests regarding the null hypothesis of equality of the index value between in 2011 and 2019.

**Table S18: Concentration index values using non-housing wealth, birth cohort 1942-1947 (for limitations in ADLs)**

|  | CI (2011) | Lower bound (2011) | Upper bound (2011) | CI (2019) | Lower bound (2011) | Upper bound (2011) | P-value |
| --- | --- | --- | --- | --- | --- | --- | --- |
| United States | -0.340 | -0.415 | -0.266 | -0.311 | -0.393 | -0.229 | 0.602 |
| Austria | -0.203 | -0.343 | -0.063 | -0.160 | -0.359 | 0.038 | 0.733 |
| Germany | -0.448 | -0.653 | -0.242 | -0.326 | -0.462 | -0.191 | 0.334 |
| Sweden | -0.187 | -0.360 | -0.014 | -0.078 | -0.226 | 0.070 | 0.347 |
| Spain | -0.193 | -0.351 | -0.035 | -0.058 | -0.239 | 0.123 | 0.271 |
| Italy | -0.365 | -0.535 | -0.194 | -0.233 | -0.410 | -0.056 | 0.294 |
| France | -0.377 | -0.523 | -0.231 | -0.018 | -0.174 | 0.139 | 0.001 |
| Denmark | -0.467 | -0.762 | -0.172 | -0.039 | -0.235 | 0.156 | 0.018 |
| Switzerland | -0.155 | -0.373 | 0.063 | -0.193 | -0.401 | 0.015 | 0.805 |
| Belgium | -0.221 | -0.336 | -0.106 | -0.224 | -0.391 | -0.058 | 0.971 |
| Czech Republic | -0.048 | -0.166 | 0.070 | -0.140 | -0.261 | -0.020 | 0.281 |
| Poland | 0.097 | -0.066 | 0.260 | -0.215 | -0.382 | -0.048 | 0.009 |
| Hungary | -0.248 | -0.396 | -0.101 | -0.457 | -0.697 | -0.216 | 0.148 |
| Slovenia | -0.224 | -0.437 | -0.011 | -0.097 | -0.256 | 0.062 | 0.348 |
| Estonia | -0.135 | -0.233 | -0.037 | -0.204 | -0.341 | -0.066 | 0.424 |
| European countries | -0.231 | -0.271 | -0.191 | -0.176 | -0.219 | -0.132 | 0.068 |

**Note:** CI is the concentration index. Lower and upper bounds refer to 95% confidence interval. P-value is derived from statistical tests regarding the null hypothesis of equality of the index value between in 2011 and 2019.

**Table S19: Concentration index values using non-housing wealth, birth cohort 1948-1953 (for limitations in ADLs)**

|  | CI (2011) | Lower bound (2011) | Upper bound (2011) | CI (2019) | Lower bound (2011) | Upper bound (2011) | P-value |
| --- | --- | --- | --- | --- | --- | --- | --- |
| United States | -0.426 | -0.482 | -0.370 | -0.389 | -0.453 | -0.324 | 0.391 |
| Austria | -0.454 | -0.603 | -0.305 | -0.516 | -0.733 | -0.299 | 0.644 |
| Germany | -0.326 | -0.523 | -0.128 | -0.260 | -0.402 | -0.117 | 0.596 |
| Sweden | -0.310 | -0.534 | -0.087 | 0.028 | -0.181 | 0.238 | 0.030 |
| Spain | -0.268 | -0.476 | -0.061 | -0.117 | -0.331 | 0.097 | 0.320 |
| Italy | -0.484 | -0.712 | -0.256 | -0.257 | -0.509 | -0.004 | 0.192 |
| France | -0.199 | -0.325 | -0.073 | -0.139 | -0.297 | 0.018 | 0.563 |
| Denmark | -0.425 | -0.681 | -0.169 | -0.139 | -0.352 | 0.074 | 0.093 |
| Switzerland | -0.453 | -0.691 | -0.215 | -0.355 | -0.601 | -0.109 | 0.575 |
| Belgium | -0.269 | -0.380 | -0.159 | -0.165 | -0.317 | -0.013 | 0.276 |
| Czech Republic | -0.173 | -0.309 | -0.037 | -0.161 | -0.292 | -0.030 | 0.899 |
| Poland | -0.179 | -0.342 | -0.017 | -0.152 | -0.299 | -0.006 | 0.810 |
| Hungary | -0.145 | -0.288 | -0.002 | -0.176 | -0.409 | 0.057 | 0.823 |
| Slovenia | -0.073 | -0.256 | 0.111 | -0.017 | -0.176 | 0.142 | 0.654 |
| Estonia | -0.187 | -0.282 | -0.093 | -0.111 | -0.253 | 0.032 | 0.381 |
| European countries | -0.264 | -0.305 | -0.223 | -0.191 | -0.237 | -0.145 | 0.021 |

**Note:** CI is the concentration index. Lower and upper bounds refer to 95% confidence interval. P-value is derived from statistical tests regarding the null hypothesis of equality of the index value between in 2011 and 2019.
